# Bayesian Variable Shrinkage and Selection in Compositional Data Regression: Application to Oral Microbiome

**DOI:** 10.1007/s41096-024-00194-9

**Published:** 2024-05-29

**Authors:** Jyotishka Datta, Dipankar Bandyopadhyay

**Affiliations:** 1 Department of Statistics, Virginia Polytechnic Institute and State University, 250 Drillfield Drive, Blacksburg, VA 24061 USA; 2 Department of Biostatistics, School of Population Health, Virginia Commonwealth University, One Capital Square, 7th Floor, 830 East Main Street, PO Box 980032, Richmond, VA 23298-0032 USA

**Keywords:** Bayesian, Compositional data, Generalized Dirichlet, Dirichlet, Large *p*, Shrinkage prior, Sparse probability vectors, Stick-breaking, Horseshoe

## Abstract

Microbiome studies generate multivariate compositional responses, such as taxa counts, which are strictly non-negative, bounded, residing within a simplex, and subject to unit-sum constraint. In presence of covariates (which can be moderate to high dimensional), they are popularly modeled via the Dirichlet-Multinomial (D-M) regression framework. In this paper, we consider a Bayesian approach for estimation and inference under a D-M compositional framework, and present a comparative evaluation of some state-of-the-art continuous shrinkage priors for efficient variable selection to identify the most significant associations between available covariates, and taxonomic abundance. Specifically, we compare the performances of the horseshoe and horseshoe+ priors (with the benchmark Bayesian lasso), utilizing Hamiltonian Monte Carlo techniques for posterior sampling, and generating posterior credible intervals. Our simulation studies using synthetic data demonstrate excellent recovery and estimation accuracy of sparse parameter regime by the continuous shrinkage priors. We further illustrate our method via application to a motivating oral microbiome data generated from the NYC-Hanes study. RStan implementation of our method is made available at the GitHub link: (https://github.com/dattahub/compshrink).

## Introduction

Human microbiome studies (Kuczynski et al. 2012) reveal microbial compositions to be linked to several human diseases, such as periodontal disease (PD; Di Stefano et al. 2022), auto-immune diseases (De Luca and Shoenfeld 2019), and cancer (Kandalai et al. 2023). A key question in this context is finding associations between taxonomic abundances of microbiomes and environmental, clinical, and/or sociodemographic predictors, while taking into account the over-dispersion and sparsity in existing associations.

Existing statistical approaches for modeling oral microbiome outcomes (our current focus) mostly rely on using a negative binomial (NB) regression model (Beghini et al. 2019; Renson et al. 2019) in conjunction with a differential abundance analysis, using the popular r packages such as edger (Robinson et al. 2010) and deseq2 (Love et al. 2014), with a false discovery rate (FDR) control mechanism to correct for multiplicity. While these approaches offer valuable insights on social inequities reflected in the oral microbiota, and identify differentially abundant operational taxonomic units (OTUs), they do not abide by the compositional framework of the responses (Gloor et al. 2017). Furthermore, they also do not engage in considering an appropriate shrinkage mechanism (on the model covariates) which can automatically adapt to sparsity, and thereby select the most important list of covariables (from an extended list). Existing shrinkage-based approaches include $\ell _1$ penalized regression on linear log-contrast models (Lin et al. 2014), or spike-and-slab priors on regression coefficients for Bayesian variable selection (Wadsworth et al. 2017). While these methods offer an ‘integrative’ view of assessing the microbiome-environment association, each of them comes with associated challenges in a fully Bayesian set-up. The convex $\ell _1$-penalties work well if one only considers the point estimator corresponding to the posterior mode or MAP estimator, however, their Bayesian analogs (e.g. a Laplace prior for Bayesian Lasso) lead to sub-optimal uncertainty quantification. In that vein, Castillo et al. (2015) pointed out that for such priors, the entire posterior distribution contracts at a suboptimal rate unlike the posterior model, and this phenomenon is driven by the insufficiently heavy tails of the Laplace prior. The spike-and-slab priors, on the other hand, often carry a heavy computational cost unless one carefully avoids the combinatorial search through the huge model space. More importantly, the idealized dichotomy of 0’s and 1’s is often deemed artificial in most applications as effect sizes are usually not exact zeroes, and relaxing this restriction leads to better performance as showcased by the continuous shrinkage priors, also called the ‘global–local’ (G-L) shrinkage priors (Bhadra et al. 2021).

Motivated by the 2013-14 NYC-HANES-II (Waldron 2023) oral microbiome data, henceforth (NYC-HANES), our objective in this paper is to quantify a precise and pragmatic relationship between the oral microbiome outcomes and a host of available sociodemographic factors. Specifically, we cast our microbiome regression framework into a Dirichlet-Multinomial (D-M) specification (Chen and Li 2013) under a Bayesian paradigm, and explore the performance of the G-L priors, specifically, the horseshoe (Carvalho et al. 2010) and horseshoe+ (Bhadra et al. 2017) priors, in terms of inducing sparsity for efficient variable shrinkage and selection, and related uncertainty assessments. The entire computational framework is powered by Hamiltonian Monte Carlo (HMC) dynamics (Betancourt et al. 2017) implemented via RStan. HMC is a Markov chain Monte Carlo (MCMC) method which adopts physical system dynamics (instead of a probability distribution considered by an usual random walk sampler) to attain the future states of the underlying Markov chain, thereby allowing a more efficient exploration of the target distribution resulting in faster convergence. A key contribution is to also produce a set of reproducible code and software in GitHub, and thus promote the generalizability of our methodology to other datasets of similar architecture.

The rest of the manuscript is organized as follows. After an introduction to the G-L shrinkage priors, our D-M regression framework in presented in Sect. 2. Section B outlines the RStan based implementation of the proposed HMC estimation scheme. The methodology is illustrated via application to the motivating NYC-HANES data in Sect. 3. The comparative efficiency of the proposed variable selection methods, and their estimation accuracy is explored via synthetic data in Sect. 4. Finally, some concluding remarks are relegated to Sect. 5, while Appendices A and B presents additional results from the motivating data analysis, and the R-Stan implementation of our modeling, respectively.

## Statistical Model

We begin with an introduction to the G-L shrinkage priors in Sect. 2.1, and then outline our D-M hierarchical model in Sect. 2.2

### Global–Local Shrinkage

The key idea behind the G-L shrinkage (Polson and Scott 2011) for high-dimensional regression is to model the parameter of interest $\varvec{\beta }$ with a prior consisting of two scale parameters: a local shrinkage parameter that helps in identifying the signals, and a global shrinkage parameter that adapts to the overall sparsity in the data. In the simplest setting, the G-L shrinkage prior is specified as follows:

2.1 $$\begin{aligned} \beta _j \sim \mathcal {N}(0, \lambda _j \tau ), \quad \lambda _j \sim f(\cdot ), \quad \tau \sim g(\cdot ) \end{aligned}$$

The hyper-priors are usually taken to be a heavy-tailed distribution that provides robustness to large signals. The parameters $\lambda _i$ and $\tau$ are called local and global shrinkage parameters, as they help identify the signals and capture sparsity, respectively. The most popular member of this family is the horseshoe estimator (Carvalho et al. 2010), which results from putting half-Cauchy priors on both *f* and *g* in (2.1). The horseshoe has inspired several continuous shrinkage priors. For example, for sharpening the signal recovery property of the horseshoe prior, Bhadra et al. (2017) proposed a horseshoe+ prior by using the novel idea of ‘Jacobian shrinkage’. The key here is an extra Jacobian term introduced in the representation on the shrinkage scale, which exhibits fundamentally different behavior for separating signals from the noise, and allows efficient signal detection in the ultra-sparse cases. There is a wealth of theoretical and practical optimality results for the class of G-L priors. We list some of them next.

(i) For the sparse normal means model, G-L priors attain the Bayes oracle risk for the multiple testing rule under a 0-1 additive loss (Ghosh et al. 2016; Datta and Ghosh 2013), and they also attain the asymptotic minimax rate for parameter estimation for ‘nearly-black’ parameter spaces (van der Pas et al. 2016), while providing excellent uncertainty quantification (van der Pas et al. 2017), at least for parameters that are either close, or away from zero.
(ii) Bhadra et al. (2016) resolve Efron’s marginalization paradoxes in Bayesian inference, by showing that the horseshoe prior can act as the default prior for non-linear functions of a high-dimensional parameter $\varvec{\beta }$. The key insight here is that the regularly varying tails of the horseshoe prior leads to robustness in presence of non-linearity.
(iii) Bhadra et al. (2019) prove that the horseshoe prior can outperform key competitors such as ridge regression in terms of *finite sample* prediction risk properties.

**Fig. 1:**
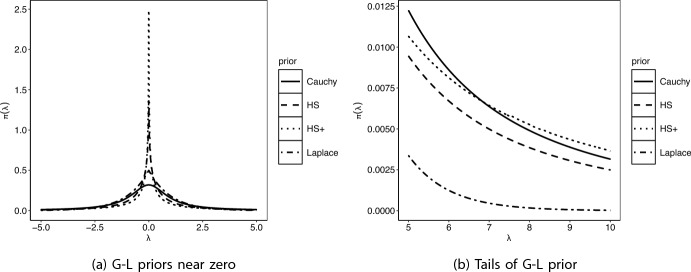
Functional forms of common G-L priors, i.e., Cauchy, Horseshoe (HS), Horseshoe+ (HS+), and Laplace, near zero (left panel), and tails (right panel). While the x-axis represents values of $\lambda$, the y-axis are the values of $\pi (\lambda )$

These priors induce sparsity but avoid the computational bottleneck of searching over an exponentially growing model space, which is often cited as the main obstacle for the implementation of the spike-and-slab class of priors on large parameter spaces. Inspired by the success of the horseshoe, many G-L priors have emerged in recent times, focusing on the sparse normal means and regression problem. Some of the popular G-L priors include the Normal Exponential Gamma (Griffin and Brown 2010), generalized double Pareto (GDP) (Armagan et al. 2013), the three-parameter beta (Armagan et al. 2011), the Dirichlet-Laplace (Bhattacharya et al. 2015), and the more recent spike-and-slab Lasso (Ročková and George 2016), horseshoe+ (Bhadra et al. 2017) and the R2-D2 (Zhang et al. 2016) priors. Figure 1 displays the functional form of some of these prior densities near the origin and their tails. As is evident from the picture, a common feature of the G-L priors is a peak near zero and heavy tails. The peak helps in adapting to sparsity while the heavy tails provide robustness to sparse signals.

It is well-known that Bayesian methods based on convex penalties, such as Bayesian Lasso, inherit the inherent problems of the associated regularization method (Castillo et al. 2015; Polson and Scott 2010). For example, the Bayesian lasso based on double exponential priors lead to non-vanishing bias in the tails due to lack of regularly varying tails. Castillo et al. (2015) argues that for Bayesian lasso, the posterior mode does not contract at the optimal rate, leading to unsatisfactory uncertainty quantification. On the other hand, the Bayesian gold-standard spike-and-slab priors work well for small to moderate dimensions, but suffers from the computational burden of searching over a combinatorial model space, untenable for huge data-dimensions. The G-L shrinkage priors (Polson and Scott 2010, 2012) is a pragmatic alternative offering an optimal solution by producing continuous scale mixture priors that can simultaneously shrink small noises to zero and leave large observations intact, while providing for fast computing strategies (e.g. Bhattacharya et al. 2016; Johndrow et al. 2020). G-L shrinkage priors are now recognized widely as the state-of-the-art Bayesian tool for shrinkage and selection, owing to their efficiency and rich theoretical support.

Our central contribution in this paper is to extend and examine the inferential capacity of these G-L shrinkage priors in terms of variable selection under a compositional regression framework, where the likelihood is characterized by the D-M distribution. We now outline the hierarchical Bayesian D-M regression framework

### Dirichlet-Multinomial Hierarchical Model with Shrinkage Prior

Consider a high-throughput sequencing data, where the total sequencing depth for each sample or location, and frequency for each of the species or taxa is known, along with a covariate vector of moderate to high dimension. Let $\textbf{y}_{i} = (y_{i1}, y_{i2}, \ldots , y_{iN})$, $1 \le i \le M$, be the vector of counts representing the abundance of different species in the $i^{th}$ sample, i.e., $y_{ij}$ is the frequency of the $j^{th}$ species from the $i^{th}$ sample. Let $\textbf{X}= (X_1, X_2, \ldots , X_p)$ be the $M \times p$ matrix of covariates. We assume the count vector for each patient/sample follows a multinomial distribution with species distribution $\varvec{\pi }_i$ for the $i^{th}$ location. The weights $\varvec{\pi }_i$ satisfies the unity-sum constraint and further follow an appropriate distribution over an *N*-dimensional simplex, such as the Dirichlet distribution.

To initiate a simple modeling framework, consider the conjugate Dirichlet prior on the compositional parameter $\varvec{\pi }_i$, i.e. each $\varvec{\pi }_i$ is given a Dirichlet prior with hyperparameter $\textbf{a}= (a_1, \ldots , a_N)$, where $a_j$’s are strictly positive shape parameters. This D-M framework is advantageous as it allows us to marginalize out the $\varvec{\pi }_i$ parameter from each step to yield the D-M likelihood, thereby offering more flexibility for count data. The D-M probability mass function for the shape parameter $\textbf{a}$ is given by:

2.2 $$\begin{aligned} p_{DM}(\textbf{y}\mid \textbf{a}) = \frac{\Gamma (\sum y + 1) \Gamma (\sum a)}{\Gamma (\sum y + \sum a)}\prod _{i = 1}^{N} \frac{\Gamma (y_j + \sum a)}{\Gamma (y_j + 1) \Gamma (\sum a)}. \end{aligned}$$

A well-known advantage of marginalizing out $\pi _i$ is that the integrated D-M model has a larger variance term to account for over-dispersion, a typical feature of most count data. Our hierarchical model is thus given by:

2.3 $$\begin{aligned}{} & {} \textbf{Y}_i \mid \pi _i \sim \text {Multinomial}(y_{i+} \mid \varvec{\pi }_i), \; y_{i+} = \sum _{j=1}^{J} y_{ij}, i = 1, \ldots , N, \nonumber \\{} & {} \quad \varvec{\pi }_i = (\pi _{i1}, \ldots , \pi _{iJ}) \mid a \sim \text {Dir}(\varvec{\pi }_i \mid a), i = 1, \ldots , N, \end{aligned}$$

where, Dir denotes the Dirichlet distribution, and *a* is the shape parameter for the Dirichlet distribution. Now, covariates can be incorporated into the setup via a logit link on the D-M shape parameter *a*, such that:

2.4 $$\begin{aligned} \eta _{ij}&= \textrm{logit}(a_{ij}), \; \eta _{ij} = \beta _{0j} + \sum _{l=1}^{p} X_{il} \beta _{lj}, \quad i = 1, \ldots , N, \nonumber \\&\beta _{lj} \sim \mathcal {N}(0, \lambda _{lj}^2 \tau _{j}^2), \; \lambda _{lj}^2 \sim \pi _{\lambda }(\tau _j), \tau _j^2 \sim \pi _{\tau }(\cdot ) . \end{aligned}$$

where, $\beta _{0j}$ is the intercept parameter, and $\beta _{lj}$ is the parameter corresponding to the covariate $X_{il}$. The parameters are given Normal priors, with various hyper-priors such as $\pi _{\lambda }(\cdot )$ and $\pi _{\tau }(\cdot )$ that induces varying shrinkage on the regression coefficients. In particular, the popular horseshoe prior (Carvalho et al. 2010) corresponds to:

$$\begin{aligned} \lambda _{lj} \sim \mathcal {C}a^+(0, \tau _j), \tau _j \sim \mathcal {C}a^+(0,1). \end{aligned}$$

where, $\mathcal {C}a^+(.,.)$ denotes the half-Cauchy density. The Bayesian Lasso corresponds to a double exponential prior (Carlin and Polson 1991; Park and Casella 2008; Hans 2009):

$$\begin{aligned} \lambda _{lj}^2 \sim \textrm{Exp}(\tau _j^2), \tau _j^2 \sim \textrm{IG}(\xi /2, \xi d^2/2) \end{aligned}$$

where, Exp(.) and $\text{ IG }(.,.)$ denotes the exponential and inverse-gamma density, respectively. Finally, for the horseshoe+ prior (Bhadra et al. 2017), one needs a product-Cauchy prior with the prior on $\lambda _{lj}$ taking the form:

$$\begin{aligned} \pi (\lambda _{lj}^2) \propto \frac{4}{\pi ^2 \tau _j}\frac{\log (\lambda _{lj}{\tau })}{(\lambda _{lj}/\tau )^2 - 1}, \end{aligned}$$

which is same as adding another layer of hierarchy:

$$\begin{aligned} \lambda _{lj} \sim \mathcal {C}a^+(0, \tau \eta _{lj}), \eta _{lj} \sim \mathcal {C}a^+(0,1). \end{aligned}$$

## Application: NYC-Hanes Data

### Data Description

The 2013-14 New York City Health and Nutrition Examination Survey, henceforth NYC-HANES-II (Thorpe et al. 2015), is a population-based survey of 1, 575 non-institutionalized adults residing in New York City. The NYC-HANES followed the design of the National Health and Nutrition Examination Survey (NHANES; NHANES 2017) conducted by the United States Centers for Disease Control and Prevention, and employed a tri-level cluster household probability sampling approach to select participants from the target population of all non-institutionalized adults, aged $\ge$ 18 years. NYC HANES-II is the second health examination survey conducted on NYC residents with a goal of assessing effect of the health policies implemented since the first NYC-HANES survey in 2004 and major changes in population health data. Analysis of sub-samples (Beghini et al. 2019; Renson et al. 2019) revealed oral microbiomes of people with varying smoking habits, diets, and oral health behaviors differed, suggesting that the oral microbiome could be a factor in health disparities. In particular, exploration of the association between tobacco exposure and oral microbiome (Beghini et al. 2019) in a sub-sample of 297 individuals revealed impaired balance of oxygen-utilizing bacteria leading to negative health outcomes. A follow-up study (Renson et al. 2019) established differential abundance of operational taxonomic units (OTU) for sociodemographic variables, such as race/ethnicity, socio-economic status (SES), marital status and others. These results shed more light on the role of microbiota as a mediator/confounder in association studies of social environment and health outcomes, although the mechanism of such differential abundance is not known yet.

**Fig. 2:**
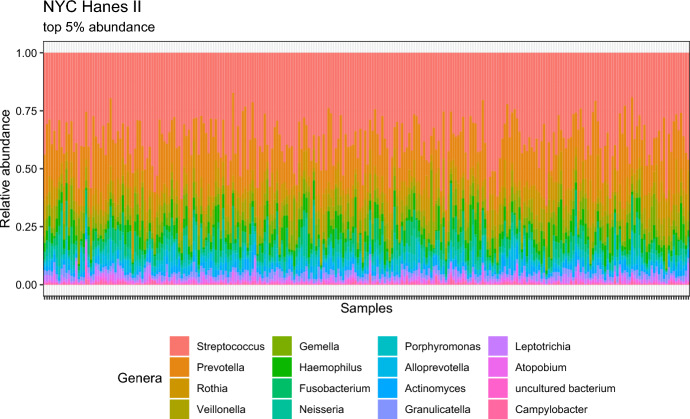
Taxononomic composition of top 5% OTUs for the NYC-Hanes Data

**Fig. 3:**
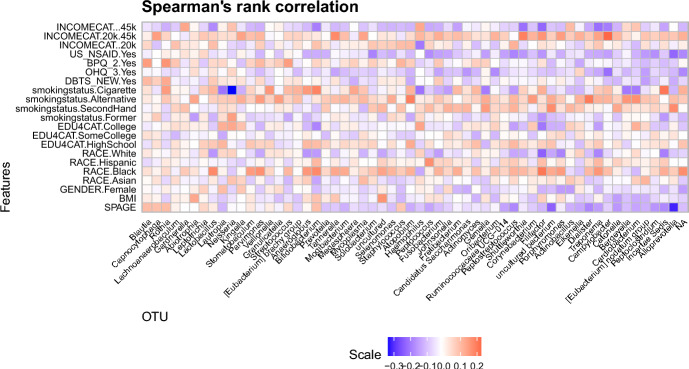
Rank Correlation heatmap for the NYC-Hanes Data

For our analysis, we analyze a sub-sample of full dataset by filtering out the rows with missing values and pregnant individuals, resulting in 264 samples and 11 sociodemographic variables (with multiple categories in several variables). The dataset and R code are available in the R package nychanesmicrobiome (Waldron 2023). We focus on the following sociodemographic variables: (a) Age (denoted as SPAGE), (b) BMI, (c) Gender (denoted as GENDER; Male, Female)), (d) Race (denoted as RACE; White, Hispanic, Black, and Asian), (e) Education categories (denoted as EDU4CAT; less than high school diploma, high school graduate/GED, some college or bachelor degree, college graduate or more), (f) Income category (denoted as INCOMECAT; $\le$ 20k, 20k-45k, and $\ge$ 45k), (g) self-reported smoking status (denoted as smokingstatus; Cigarette, Alternative, SecondHand, and Former), (h) Diabetes, based on FPG, HbA1C, or self-report (denoted as DBTS-NEW; Yes/No), (i) Answer to the survey question, "does respondent think respondent has gum disease?" (denoted by OHQ3; Yes/No), (j) Answer to the survey question, “Has SP ever been told by a doctor or other health professional that SP had hypertension, also called high blood pressure?" (denoted by BPQ2; Yes/No), and (k) Answer to the survey question, “Have you taken any aspirin, steroids, or other non-steroidal anti-inflammatory medicine in the past week?” (denoted by US-NSAID; Yes/No). Our response variable read count is at the genus level, limited to genera present in $>20\%$ samples, resulting in 64 distinct OTUs. The taxonomic composition of the present subsample is similar to what is reported in previous papers (Beghini et al. 2019), in particular, the dominant genera are *Streptococcus* and *Prevotella*, with other commonly found genera, such as *Rothia*, *Veillonella* and *Gemella*. Figure 2 presents the taxonomic composition heatmap of the top 5% OTUs in the data, while Fig. 3 shows the Spearman’s rank correlation heatmap between the sociodemographic variables and all the 64 genera present. This correlation plot suggests quantifiable (marginal) relationship between the variables and the OTUs.

### Model Fitting and Findings

**Fig. 4:**
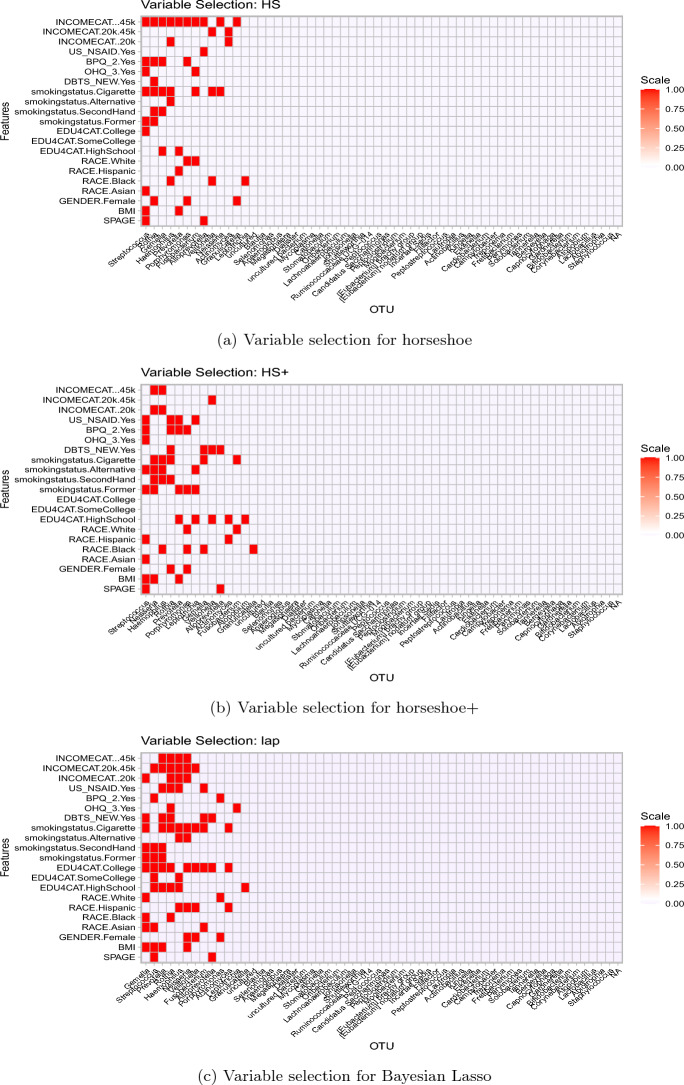
Selected $\beta _{i,j}$’s from fitting the three competing methods to the NYC-Hanes data

We now fit the D-M hierarchical shrinkage model to the NYC-Hanes data with three candidate priors: horseshoe (Carvalho et al. 2010), horseshoe+ (Bhadra et al. 2017) and the Bayesian Lasso (Park and Casella 2008). We use the popular stan software to implement posterior sampling, as described in Section B. For selecting non-zero associations, we use a criterion of 95% credible intervals for $\beta _{ij}$ containing zero, although other methods could be used, such as the 2-means strategy by Li and Pati (2017). As expected, horseshoe and horseshoe+ leads to a sparser association recovery than Bayesian Lasso (or Laplace); the number of selected $\beta$’s by the above criteria for the three priors were horseshoe: 51, horseshoe+: 56 and Bayesian Lasso: 79. Figure 4 displays the selected $\beta _{ij}$’s obtained from the competing methods; these can be interpreted as the recovered associations between the socio-demographic variables and the genera in the NYC-Hanes data. Figure 4 also shows both the sparsity patterns as well as the commonality of the performances of the shrinkage priors. As noted earlier, the Bayesian Lasso tends to select more associations due to its less aggressive shrinkage near zero, as opposed to horseshoe-type priors with a spike at the origin.

**Table 1:** Selected microbial genera by the three candidate shrinkage priors using 95% Credible Intervals for cigarette smokers (reference level set to non-smoker). The mean column refers to the estimated posterior mean $\hat{\beta }_{ij}$

Smoking Status: Cigarette Baseline: Non-smoker					
Bayes-Lasso	Mean	HS	Mean	HS+	Mean
Fusobacterium	$-$3.43475	Fusobacterium	$-$0.10024	Fusobacterium	$-$0.12071
Gemella	$-$3.93223	Gemella	$-$0.15	Gemella	$-$0.24661
Haemophilus	$-$0.17698	Haemophilus	$-$0.14241	Haemophilus	$-$0.23351
Neisseria	$-$3.16806	Neisseria	$-$3.17861	Neisseria	$-$3.20674
Rothia	0.118395	Rothia	0.116904	Rothia	0.069546
Veillonella	0.151276	Streptococcus	0.027135		
Actinomyces	0.10477	Veillonella	0.139699		
Prevotella	0.056836				

We now interpret the selected genera corresponding to the smoking status variable. Table 1 presents the selected genera, when ‘smokingstatus = cigarette’ (with non-smokers as baseline). We observe that *Prevotella*, *Haemophilus*, and *Neisseria* are among the selected genera; these were also identified in earlier work (Beghini et al. 2019). In smokers, a reduction in Bacteroides and Proteobacteria were observed, accompanied by a decrease in genera also identified by this model, for example, *Prevotella*, *Haemophilus*, *Neisseria* etc., aligning with findings from prior studies (Charlson et al. 2010; Morris and Tang 2011; Wu et al. 2016). Morris and Tang (2011) compared microbial composition in lung and oral cavity for 64 individuals, and noted that while the microbial communities resemble each other between lung and oral cavity, there exists notable differences, and in particular, the distribution of *Neisseria*, *Gemella* and *Porphyromonas* differed in the oral cavity of smokers versus non-smokers. Notably, the diminished presence of *Proteobacteria* appears to be significant, given its association with PD observed in individuals compared to their healthy counterparts (Griffen et al. 2012). Charlson et al. (2010) reported that smoking leads to a simultaneous reduction in commensal (harmless) microbes (e.g. *Prevotella*) and increase or enrichment of potential pathogens like *Streptococcus* and *Haemophilus*. The reduced abundance of normal oral microbiota, such as *Fusobacterium* and *Neisseria* in smokers compared to non-smokers, as reported by Charlson et al. (2010), is aligned with the negative associations reported in our integrated DM model (see, Table 1). Additional results on the selected microbial genera by the three candidate priors corresponding to the BMI and Gender predictors are presented in Appendix A.

### Multivariate Posterior Predictive Model Assessment

Posterior predictive p-values (Meng 1994; Gelman et al. 1996, 2014) are often used as the primary diagnostic tool for model assessment, under the Bayesian paradigm. The underlying intuition here is that if the model fits the data well, then the original observations will be ‘similar’ to the replicates from the posterior distribution under the model. More formally, if $p(y^{rep} \mid y)$ denotes the posterior predictive distribution given by: $p(y^{rep} \mid y) = \int p(y^{rep} \mid \Theta ) p(\Theta \mid y) d\Theta$, then the posterior predictive p-value (PPP) is given by $p(T(y^{rep} > T(y) \mid y)$, where the integration is with respect to the posterior predictive distribution $p(y^{rep} \mid y)$. A PPV value close to 0 or 1 is indicative of a poor fit.

However, in this paper, the response variable $\textbf{y}$ lies in a *K* dimensional simplex $\S _K$, and a suitable metric for model checking that accounts for multivariate outcomes is needed. We adopt a natural extension of the posterior predictive model checking to multivariate outcomes, proposed by Crespi and Boscardin (2009) using dissimilarity measures. Here, the primary idea is to calculate pairwise distances between the observed and the posterior predictive replicates (say $d^{obs}_{j} = d(\textbf{y}^{obs}, \textbf{y}_j^{rep})$, for $j = 1, \ldots , J$) as well as pairwise distances between the posterior predictive replicates (say $d^{rep}_{ij} = d(\textbf{y}_i^{rep}, \textbf{y}_j^{rep})$, for $i \ne j = 1, \ldots , J$), producing two sets of dissimilarity measures. Then, if the model captures the multivariate $\textbf{y}$ well, the values $d^{rep}_{ij}$ and $d^{obs}_j$ should be stochastically similar.

**Fig. 5:**
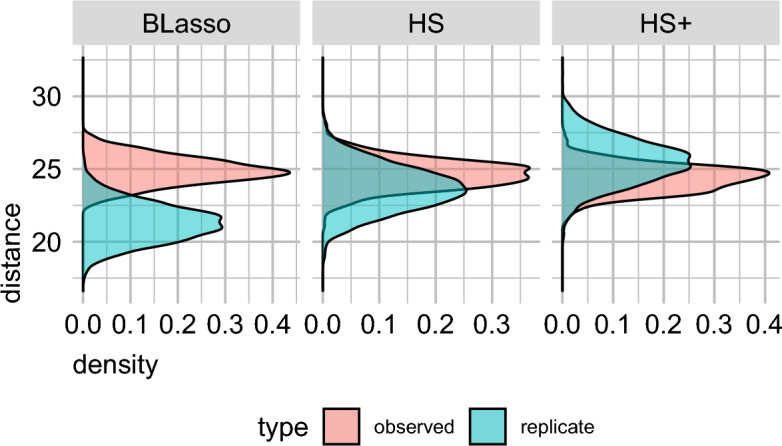
Density plots of the dissimilarity measures corresponding to the horseshoe, horseshoe+, and Laplace prior (Bayesian Lasso) assumptions in our proposed D-M model

Figure 5 presents the posterior density plots for the observed and replicate dissimilarity measures for the three candidate priors: horseshoe, horseshoe+ and Bayesian Lasso (or, Laplace) in the D-M model. The plots reveal that the degree of overlap is much smaller for the Laplace prior compared to the two other shrinkage priors, indicating better fit for the latter methods.

## Simulation Studies

In this section, we evaluate the finite sample performances of our proposed method in terms of the accuracy of variable selection, as well as comparisons between the horseshoe, horseshoe+, and Bayesian lasso prior assumptions via simulation studies with synthetic data.

### Scheme I(a): Evaluation of Finite-Sample Performance and Estimation Accuracy

To evaluate the variable selection performance of model (2.3) – (2.4), we generate the true $\beta$ from a mixture of uniforms and a point mass at zero, given by $\beta _{jl} \sim \pi ~U(L,U) + (1-\pi )~ \delta _{\{0\}}$. We draw *X* from a multivariate Normal, such that $X \sim \mathcal {N}(0, \Sigma )$, $\Sigma = \rho ^{|i-j|}$, and $\rho = 0.4$ as in Wadsworth et al. (2017). The count matrix *Y* is generated from a multinomial distribution, i.e., $Y_i \sim \text {Multinomial}(y_{i+}, p_i)$, where row-total $y_{i+}$ follows a discrete uniform $\text {Unif}(\{1000, 5000\})$. Finally, $\varvec{\pi }_i$ follow a Dirichlet distribution with an overdispersion parameter $\psi$, given by $\varvec{\pi }_i \sim \text {Dir}\left( \frac{\psi }{1-\psi } (a_{i1}, \ldots , a_{iJ}) \right)$, where the probabilities are linked to the predictor variables as: $p_{i} = \text {softmax}(\eta \frac{\psi }{1-\psi }),$ where $\text {softmax}(z) = \exp (\textbf{z})/\sum (\exp (\textbf{z}))$ and $\eta = X\beta$.

**Fig. 6:**
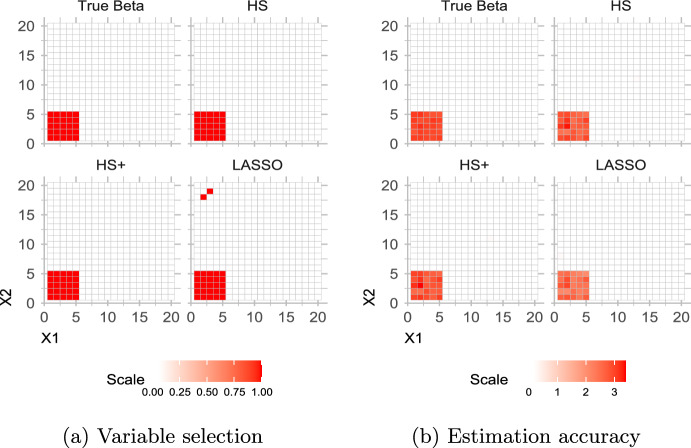
Simulation scheme I(a) results, evaluating recovery of true non-zero associations (left panel), and estimation accuracy (right panel)

We first examine the performance of the horseshoe prior vis-a-vis horseshoe+ and Bayesian Lasso prior. For this simulation study, a single model fit incorporates four chains with 2000 iterations each, where first 1000 iterations of each chain are discarded as warm-up leaving in total, 4000 post-warm-up samples. We observed that a smaller sample size and fewer chains make no qualitative difference to the posterior estimates. The number of observations, number of predictors and number of taxa are fixed at $n = 50$, $p = 20$ and $q = 20$ respectively, leading to the number of estimable parameters in $\varvec{\beta }$ matrix to be $p \times q = 400$ (which is more than the number of observations). We also set the number of relevant predictors ($p_0$) and number of relevant taxa ($q_0$) both at 5, i.e. the proportion of non-zero parameters in $\varvec{\beta }$ is $p_0q_0/pq = 0.0625$. This set-up is chosen to induce sparsity in the generating model. Figure 6 (left panel) suggests that we can recover the true non-zero associations from the data, and the posterior inclusion probability concentrates to a higher value for the true association values. The two red dots indicate false discoveries, as expected, by the Bayesian lasso. For the same data, we compare the estimation accuracy performances of the candidate variable selection methods via 95% posterior credible intervals. This was done following the suggestions in van der Pas et al. (2016); Wei (2017); Tadesse and Vannucci (2019), where variable selection is performed by checking whether the posterior credible interval contains zero or not, at a nominal level. As shown in Fig. 6 (right panel), the Bayesian Lasso misses two of true non-zero $\beta$’s (corresponding to the two red dots on the left panel of Fig. 6), but correctly shrinks all null $\beta$’s to zero. This is expected, since the Bayesian Lasso does not shrink to zero as strongly as horseshoe or horseshoe+, because of its lack of large prior mass at zero (Polson and Scott 2010).

### Scheme I(b): Evaluation of Variable Selection Strategies

**Fig. 7:**
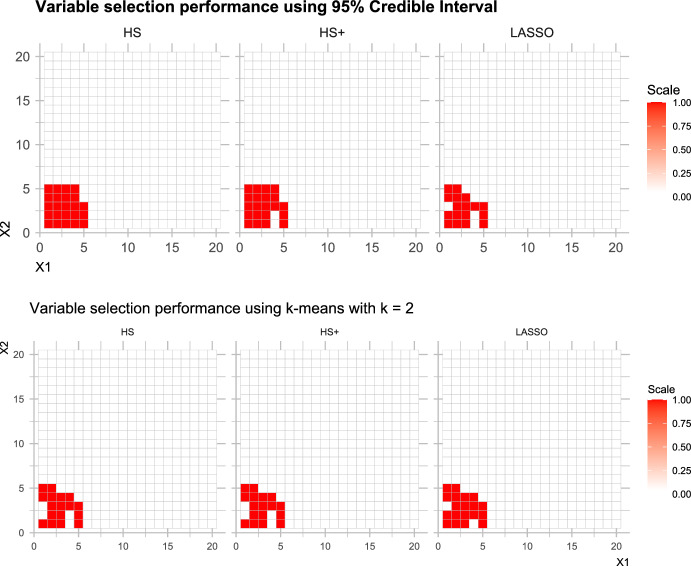
Simulation scheme 1(b) results, comparing recovery of true non-zero associations, via the 95% credible interval method (upper panel), and the 2-means model (lower panel)

As suggested by a referee, we now compare the 95% credible interval (CrI) approach for variable selection with the two-means (2 M) algorithm as used by Bhattacharya et al. (2015). We follow the simulation set-up as Scheme I above, but under a weaker correlation $\rho = 0.1$. Figure 7 shows the variable selection performance for the 95% CrI approach alongside the 2 M approach. It seems that the 2 M approach performs worse than the CrI approach for all but the Bayesian Lasso. In particular, out of the 25 true non-zero entries in $\varvec{\beta }$, the horseshoe prior selects the maximum number (23) of non-zero associations using the 95% CrI approach, as shown in Table 2. It is interesting to note that none of the candidate priors result in any false discoveries.

**Table 2:** Simulation scheme 1(b) results, where table entries denote the number of non-zero associations detected via the posterior 95% credible interval method, and the 2-means clustering method. The number of true non-zero entries in $\varvec{\beta }$ were 25

Method	Horseshoe	Horseshoe+	Bayes Lasso
95% Credible Interval	23	21	17
2-means clustering	17	19	19

While there is no consensus over a single established method for variable selection with continuous shrinkage priors, several approaches exist to identify the final set of relevant variables. These include:

(i) Credible intervals (at a pre-defined nominal level) covering zero as suggested by van der Pas et al. (2017) or Tadesse and Vanucci (Tadesse and Vannucci 2019). This is a more conservative (few discoveries) and ideal for minimizing false positives. We have used this approach for variable selection.
(ii) Thresholding shrinkage factors as in Tang and Chen (2018): This is limited to scenarios where the number of variables (*p*) is less than the number of samples (*n*), and, to our knowledge, only defined in terms of linear regression and sequence model.
(iii) Decoupling shrinkage and selection (DSS) (Hahn and Lopes 2014): This method aims to create a sparse posterior mean that explains most of the predictive variability. However, its focus on prediction might not be optimal for problems estimating regression coefficients with correlated regressors.
(iv) Penalized credible regions by Zhang et al. (2016): This approach seeks the sparsest model within a specific credible region, viz., the $100\times (1-\alpha )$% joint elliptical credible region.
(v) Two-means clustering, used in Bhattacharya et al. (2015), and further investigated by Li and Pati (2017). This approach proceeds via post processing of the posterior samples. Initially, a posterior distribution of the number of signals is obtained by clustering the signal and the noise coefficients, which eventually leads to estimating the signals from the posterior median. While this remains an attractive method due to its generalist and tuning-free nature, it may not always be reliable for consistent selection, as in our case, shown above.

Additionally, if a sparse point estimator or variable selection is the primary goal, using the posterior mode estimator with the horseshoe prior could be a more suitable strategy as it leads to exact zeros for selected variables. For joint posterior mode calculation with horseshoe-like priors, an approximate algorithm was proposed in Bhadra et al. (2021). A thorough comparison of all the variable selection strategies for a D-M model would be an interesting future study.

### Scheme II: Comparison Between shrinkage Priors

Next, we compare the performance of the three candidate shrinkage priors, *viz.* horseshoe, horseshoe+ and Bayesian Lasso. There is now a large and growing list of continuous shrinkage priors for inducing sparsity in parameter estimates, and we chose these priors as they offer state-of-the-art solution without the need for tuning any hyper-parameters. We also excluded spike-and-slab priors or its variants or mixtures from this comparison as our goal is producing posterior samples in reasonable time, and not just obtaining a point-estimate using an EM-type algorithm.

**Fig. 8:**
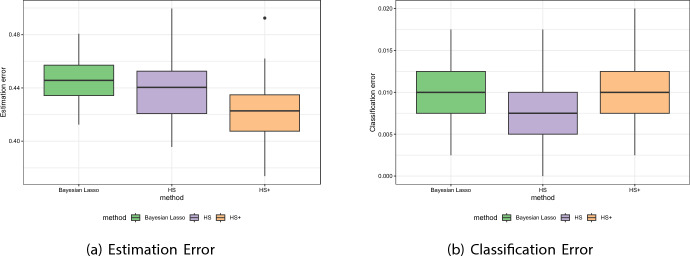
Simulation scheme II results, presenting boxplots of estimation (left panel) and misclassification (right panel) errors, corresponding to the three shrinkage priors, i.e., horseshoe, horseshoe+ and Bayesian Lasso

The simulation design here mimics Scheme I, yet, under weak dependence assumption between the predictors, i.e. the correlation coefficient is now fixed at $\rho = 0.1$. We compare the estimation and classification accuracies for the three candidate priors over 50 replicated datasets, where the accuracy of an estimator is measured as $\Vert \hat{\varvec{\beta }} - \varvec{\beta }_{0}\Vert _{F}/\Vert \varvec{\beta }_0\Vert _{F}$, with $\Vert \textbf{x}\Vert _F$ as the Frobenius norm of $\textbf{x}$. Figure 8 present boxplots of estimation (left panel) and misclassification (right panel) errors, corresponding to the three shrinkage priors, while Table 3 presents summary statistics (mean, median and standard deviation) corresponding to Fig. 8. We observe that horseshoe+ beats both horseshoe and Bayesian Lasso in terms of estimation (the error mean and median for the horseshoe+, although closer to the horseshoe, is the lowest, and much lower than the Bayesian Lasso), while the horseshoe outperforms the others (having the lowest mean and median error estimates) in regards to misclassification error. This seems to be an outcome of the fact that the horseshoe induces a non-convex penalization on the sparse parameter vector unlike the Bayes Lasso (Bhadra et al. 2021), and this non-convexity protects against both low signal-to-noise ratio as well as predictor dependence (Mazumder et al. 2012).

**Table 3:** Simulation scheme II results, presenting summary statistics (mean, median and standard deviation) of estimation (left panel) and classification (right panel) errors, corresponding to the three competing shrinkage priors, i.e., horseshoe prior, horseshoe+ prior and Laplace prior

(a) Estimation Error			
Prior	Mean	Median	SD
Bayes Lasso	0.447	0.446	0.0167
HS	0.438	0.440	0.0221
HS+	0.421	0.423	0.0254

(b) Classification Error			
Prior	Mean	Median	SD
Bayes Lasso	0.0102	0.010	0.00332
HS	0.0084	0.0075	0.00342
HS+	0.0105	0.010	0.00315

## Conclusions

In this paper, we presented a Bayesian inferential approach to the D-M compositional regression model with horseshoe, horseshoe+ and Bayesian lasso prior choices for efficient variable selection. with illustration via application to the NYC-Hanes II oral microbiome data. We also performed a simulation study to compare the relative performances of the three priors, in terms of true signals recovery. We observe that both the horseshoe and horseshoe+ priors outperform the Bayesian Lasso. While a theoretical investigation is beyond scope of this paper, we plan to take this up on a future endeavor. Our conjecture is that the heavy tails of global–local shrinkage priors coupled with the spike at zero are responsible for the superior performance, compared to the Bayesian Lasso.

There have been some attempts to generalize the Dirichlet distribution to yield a more flexible and rich parametric family containing the simple Dirichlet. For example, Connor and Mosimann (1969) introduced the generalized Dirichlet (GD) distribution to yield a more flexible covariance structure while maintaining conjugacy, thereby making it more practical and useful (see, Kotz et al. 2000, pp. 520-521) and Wong (1998). The GD distribution is as follows:

5.1 $$\begin{aligned} Z_{j}&\sim {\text {Beta}}\left( a_{j}, b_{j}\right) , j=1, \ldots , K \nonumber \\ \pi _{j}&=Z_{j} \prod _{i=1}^{j-1}\left( 1-Z_{i}\right) \Rightarrow Z_{j}=\pi _{j} /\left( 1-\sum _{i=1}^{j-1} \pi _{i}\right) \nonumber \\ \mathbf {\varvec{\pi }}&=\left( \pi _{1}, \ldots , \pi _{K}\right) \sim {\text {GD}}({\textbf{a}}, {\textbf{b}}) \end{aligned}$$

The Dirichlet distribution can be derived as a special case of the GD distribution if $b_{j-1} = a_j + b_j$; in particular, the symmetric Dirichlet density $\text {Dir}(\alpha /K, \ldots , \alpha /K)$ results if $a_j = \alpha /K, b_j = \alpha (1-j/K)$. The GD distribution has a more general covariance structure compared to the Dirichlet, and it maintains the nice properties of Dirichlet, such as conjugacy to multinomial likelihood and complete neutrality (Connor and Mosimann 1969). An alternative parametric form to (5.1) proposed by Wong (1998) is as follows:

5.2 $$\begin{aligned} f(\varvec{\pi })&= \prod _{i=1}^{K}\frac{1}{\text {Be}(\alpha _i, \beta _i)} \pi _i^{\alpha _i - 1} (1- \pi _1 - \cdots - \pi _i)^{\gamma _i} \nonumber \\&\text {for} \; \pi _i \ge 0, \text { and } \sum _{i=1}^{K} \pi _i = 1, \gamma _i = \beta _i - \alpha _{i+1} - \beta _{i+1}, i = 1, \ldots , K -1. \end{aligned}$$

From (5.2), it is obvious that imposing symmetry via $\gamma _i = 0$ reduces the Connor–Mosimann construction (5.1) to the Dirichlet distribution.

A natural extension of (2.3) – (2.4) is to use the GD prior to model the simplex-valued $\varvec{\pi }_i$. Under the GD prior, our hierarchical model would become:

5.3 $$\begin{aligned}{} & {} \textbf{Y}_i \mid \varvec{\pi }_i \sim \text {Multinomial}(y_{i+} \mid \ \varvec{\pi }_i), \; y_{i+} = \sum _{j=1}^{J} y_{ij}, i = 1, \ldots , N,\nonumber \\{} & {} \quad \varvec{\pi }_i = (\pi _{i1}, \ldots , \pi _{iJ}) \mid \textbf{a}, \textbf{b}, \varvec{\phi }\sim {\text {GenDir}}(\varvec{\pi }_i \mid \textbf{a}, \textbf{b}, \varvec{\phi }), i = 1, \ldots , N. \end{aligned}$$

Then, we incorporate the covariates into the GD model using a log-linear regression approach via the log-shape parameter. Our hierarchical model is thus given as:

5.4 $$\begin{aligned}{} & {} \textbf{Y}_i \mid \varvec{\pi }_i \sim \text {Multinomial}(y_{i+} \mid \varvec{\pi }_i), \; y_{i+} = \sum _{j=1}^{J} y_{ij}, i = 1, \ldots , N,\nonumber \\{} & {} \quad \varvec{\pi }_i = (\pi _{i1}, \ldots , \pi _{iJ}) \mid \textbf{a}, \textbf{b}, \varvec{\phi }\sim \text{ GD }(\varvec{\pi }_i \mid \textbf{a}, \textbf{b}), i = 1, \ldots , N, \nonumber \\{} & {} \quad \mu _{ij} = \frac{\textrm{e}^{\varvec{\beta }_j^T \textbf{x}_i}}{1+\textrm{e}^{\varvec{\beta }_j^T \textbf{x}_i}}, \sigma _{ij} = \frac{\textrm{e}^{\varvec{\alpha }_0}}{1+\textrm{e}^{\varvec{\alpha }_0}}, \nonumber \\{} & {} \quad \beta _{lj} \sim \pi _{\lambda }(0, \lambda _{lj}^2 \tau _{j}^2), \; \lambda _{lj}^2 \sim \pi _{\tau }(\tau _j), \tau _j^2 \sim \sim \pi _0(\cdot ), \nonumber \\{} & {} \quad \alpha _{0j} \sim \pi _{\alpha }(\tau _0), {\tilde{\tau }}_0 > 0. \end{aligned}$$

A potential issue with the GD modeling framework is over-parametrization; the GD distribution has almost twice as many parameters compared to a typical Dirichlet distribution. However, there exists other extensions of the GD regression framework, such as the zero-inflated GD (ZIGD; Tang and Chen 2018) regression, which has been proposed as a flexible alternative to handle the presence of excess ‘structural’ zeroes among the multivariate taxa counts in compositional data regression. Under the Bayesian paradigm, exploring and comparing the efficiency of the family of continuous shrinkage priors (to the usual spike-and-slab alternatives) now under the GD and ZIGD frameworks are credible extensions. Also, in microbiome studies, relative abundance of species could vary with time, and considering the effect of time within a longitudinal compositional regression framework seems worthwhile. However, such dynamic modeling will require additional modification to the Bayesian variable selection strategy proposed here. These will be pursued elsewhere.

## NYC-Hanes Data Application: Additional Results

Here, we present the selected clinically important associations for two other important predictors viz., Body-Mass Index (BMI) and Gender. As Table 4 shows, *Prevotella*, and *Streptococcus* were among the selected genera for association with BMI for all three candidate priors. On the other hand, for ‘gender = female’, *Porphyromonas* was the only selected genera across all priors, and *Rothia* was present for both horseshoe and horseshoe+. *Streptococcus* and *Prevotella* were also among the most abundant genera, as reported by Renson et al. (2019).

**Table 4:** Selected microbial genera by the three candidate shrinkage priors using 95% Credible Intervals for BMI and Gender (reference level set to male). The mean column refers to the estimated posterior mean $\hat{\beta }_{ij}$

Bayes-Lasso		HS+		HS	
BMI		BMI		BMI	
Gemella	0.069933	Neisseria	$-$0.094633034	Prevotella	0.052712
Neisseria	$-$0.16766	Prevotella	0.089436593	Streptococcus	$-$0.02665
Prevotella	0.073241	Streptococcus	$-$0.040095512		
Streptococcus	$-$0.05538				
Gender.Female		Gender.Female		Gender.Female	
Neisseria	$-$0.0823	Porphyromonas	$-$0.233645618	Granulicatella	1.773207
Porphyromonas	$-$0.251	Rothia	0.093621631	Porphyromonas	$-$0.22347
Veillonella	0.071075			Rothia	0.119202

## R-Stan Implementation

stan(Carpenter et al. 2017) is an efficient probabilistic programming language for specifying and fitting complex statistical models under a Bayesian paradigm. One of stan’s key strengths lies in its ability to efficiently handle both small and large datasets, enabling users to specify and fit a wide range of statistical models. stan  uses the No-U-Turn Sampler (NUTS) for HMC sampling, which is a state-of-the-art algorithm that often outperforms traditional methods, such as Gibbs sampler or Metropolis–Hastings for complex posteriors (Neal 2011) in terms of speed and accuracy. stan  also supports a wide variety of other computational tools, such as variational Bayes or expectation propagation by providing easy access to log-densities and their gradient, hessians and related quantities. With a growing community of users and extensive documentation, as well as interfaces with most programming languages (such as rstan or pystan, stan  has become a popular choice for researchers, data scientists, and statisticians who seek a powerful and user-friendly tool for tackling challenging statistical problems using probabilistic programming. Here, we provide the outline for implementing the D-M model in stan  in the spirit of open-source programming. The r as well as stan  implementations for other candidate shrinkage priors are available at the GitHub link: https://github.com/dattahub/compshrink.

**Horseshoe prior:** We first show the stan implementation for the Bayesian hierarchical integrated D-Mmodel, as specified in equations (2.3) – (2.4), with the horseshoe prior for variable selection. The stan  program below is typically divided into four main blocks: data, parameters, transformed parameters, and model. The first optional block functions defines user-defined functions, and we have defined the marginalized D-M distribution here that one can obtain by integrating out $\varvec{\pi }$.

**Horseshoe+ prior:** The horseshoe+ prior implementation in stan  is very similar to the horseshoe implementation above, and we have omitted the data chunk as it is identical as above. We use non-centered parametrization (Papaspiliopoulos et al. 2007) using the optional transformed parameters block to reduce correlation between lower-level parameters and increase efficiency in presence of relatively smaller sample size compared to model dimensions. To do this, we define $\tilde{\lambda }$ (lambda_tilde) as the parameter with a $\mathcal {C}a^+(0,1)$ prior and define $\lambda$ (lambda) as $\lambda = \tilde{\lambda } \eta$.

**Bayesian Lasso or Laplace prior:** Finally, we present the stan  code for the Bayesian Lasso (Park and Casella 2008; Hans 2009), without the data chunk. Similar to the other shrinkage priors used here, we use a non-centered parametrization.
